# Investigation of Four Clusters of Severe Acute Respiratory Syndrome Coronavirus 2 (SARS-CoV-2) in Rwanda, 2020

**DOI:** 10.3390/ijerph18137018

**Published:** 2021-06-30

**Authors:** Olivier Nsekuye, Edson Rwagasore, Marie Aime Muhimpundu, Ziad El-Khatib, Daniel Ntabanganyimana, Eric Noël Kamayirese, Laurent Ruyange, Angela Umutoni, Adeline Kabeja Adeline, Joseph Ntaganira, Sabin Nsazimana, Jared Omolo

**Affiliations:** 1 Blood Transfusion Division, Rwanda Biomedical Center (RBC), Kigali 7162, Rwanda; 2 Rwanda Field Epidemiology and Laboratory Training Program, Department of Epidemiology and Biostatistics, University of Rwanda, Kigali 3286, Rwanda; ntabadaniel@gmail.com (D.N.); kaericnoel@gmail.com (E.N.K.); ruyangelaurent@gmail.com (L.R.); jntaganira@nursph.org (J.N.); wtq0@cdc.gov (J.O.); 3 Public Health Surveillance and Epidemic, Rwanda Biomedical Center (RBC), Kigali 7162, Rwanda; Edson.rwagasore@rbc.gov.rw (E.R.); mmuhimpundu@gmail.com (M.A.M.); angelatoni01@gmail.com (A.U.); adelinekabeja@gmail.com (A.K.A.); 4 Bill and Joyce Cumming Institute of Global Health, University of Global Health Equity, Kigali 6955, Rwanda; ziad.khatib@gmail.com; 5 Department of Global Public Health, Karolinska Institutet, 17177 Solna, Sweden; 6 World Health Programme, Université du Québec en Abitibi-Témiscamingue, Rouyn-Noranda, QC J9X 5E4, Canada; 7 Rwanda Biomedical Center (RBC), Kigali 7162, Rwanda; sabin.nsanzimana@rbc.gov.rw

**Keywords:** COVID19, transmission, Rwanda, infectious diseases, cluster analysis

## Abstract

We reported the findings of the first Severe Acute Respiratory Syndrome Coronavirus 2 (SARS-CoV-2) four clusters identified in Rwanda. Case-investigations included contact elicitation, testing, and isolation/quarantine of confirmed cases. Socio-demographic and clinical data on cases and contacts were collected. A confirmed case was a person with laboratory confirmation of SARS-CoV-2 infection (PCR) while a contact was any person who had contact with a SARS-CoV-2 confirmed case within 72 h prior, to 14 days after symptom onset; or 14 days before collection of the laboratory-positive sample for asymptomatic cases. High risk contacts were those who had come into unprotected face-to-face contact or had been in a closed environment with a SARS-CoV-2 case for >15 min. Forty cases were reported from four clusters by 22 April 2020, accounting for 61% of locally transmitted cases within six weeks. Clusters A, B, C and D were associated with two nightclubs, one house party, and different families or households living in the same compound (multi-family dwelling). Thirty-six of the 1035 contacts tested were positive (secondary attack rate: 3.5%). Positivity rates were highest among the high-risk contacts compared to low-risk contacts (10% vs. 2.2%). Index cases in three of the clusters were imported through international travelling. Fifteen of the 40 cases (38%) were asymptomatic while 13/25 (52%) and 8/25 (32%) of symptomatic cases had a cough and fever respectively. Gatherings in closed spaces were the main early drivers of transmission. Systematic case-investigations contact tracing and testing likely contributed to the early containment of SARS-CoV-2 in Rwanda.

## 1. Introduction

In late December 2019, an alert about a group of cases presenting with pneumonia of unknown etiology was reported to the World Health Organization (WHO) by the local authorities in China [1]. The causative agent was subsequently confirmed to be a novel corona virus that WHO named Severe Acute Respiratory Syndrome Coronavirus 2 (SARS-CoV-2) and the disease as coronavirus disease 2019 (COVID-19) [2]. Declared a public health emergency of international concern by WHO on 30 January 2020, the COVID-19 pandemic has since evolved into a major global health disaster with severe public health and economic impact. Globally, over 4 million cases and close to 300,000 deaths had been reported within 100 days of WHO’s declaration. Within the same period, as of 11 May 2020, the WHO AFRO Region had reported 44,533 cases and 1425 deaths [3].

Africa reported its first confirmed case of COVID-19 in Egypt on 14 February 2020 [4]. In Rwanda, the Ministry of Health reported the country’s first case of COVID-19 on 14 March 2020 [5]. Within the first forty days (14 March–22 April), a total of 154 COVID-19 cases confirmed by laboratory (PCR) with no deaths had been reported. Although most of the early cases were imported from countries where transmission had been established, community transmission could not be ruled out and the country initiated an in-depth epidemiological investigation, quarantining and testing of contacts, and isolation of confirmed cases in order to contain the disease. With the identification of close linkages among the early cases, these detailed investigations became a key component in informing the country’s early response strategy. We reported the findings from the epidemiological investigations that were conducted in Rwanda within the first six weeks of the pandemic (14 March to 22 April 2020) and informed the initial response measures by helping to identify four clusters early in the outbreak.

## 2. Materials and Methods

### 2.1. Case Identification

Upon the detection of the first case of COVID-19, Rwanda activated a multi-disciplinary task force to guide response and instituted a number of prevention and control measures to mitigate the impact. These included establishment of social distancing measures like lockdown (14 March to 4 May 2020), active case finding, isolation and clinical care of confirmed cases and quarantining and testing of contacts.

In Rwanda, early surveillance approaches included screening and testing of people arriving from countries where COVID-19 had already been reported. In addition, patients presenting in health facilities with a compatible case definition were also tested as were health care workers and other frontline staff identified as high risk.

A confirmed case was defined as a person with laboratory confirmation on Reverse Transcription-Polymerase Chain Reaction (RT-PCR) test of COVID-19 infection, irrespective of clinical signs and symptoms.

A suspect case was defined as a patient with any of the following symptoms: (i) an acute respiratory illness (fever and at least one sign/symptom of respiratory disease, e.g., cough, shortness of breath), and a history of travel to or residence in a location reporting confirmed case(s) of COVID-19 disease during the 14 days prior to symptom onset; (ii) or a patient with any acute respiratory illness (fever and at least one sign/symptom of respiratory disease, e.g., cough, shortness of breath) and having been in contact with a confirmed COVID-19 case in the previous 14 days prior to symptom onset; (iii) or a patient with severe acute respiratory illness (fever and at least one sign/symptom of respiratory disease, e.g., cough, shortness of breath) and in the absence of an alternative diagnosis that fully explains the clinical presentation.

### 2.2. Epidemiological Investigations

An in-depth epidemiological investigation for all confirmed cases were conducted by field epidemiologists from the Rwanda Field Epidemiology Program and Rwanda Biomedical Centre (RBC). Confirmed cases were interviewed to get information on demographic characteristics, clinical signs and symptoms and their activities in the past three weeks. Contacts were identified and classified into categories depending on the contact setting, the type of contact and the length of the contact event. A contact of a COVID-19 case was defined as any person who had contact with a COVID-19 case within a timeframe ranging from 72 h before the onset of symptoms of the case to 14 days after the onset of symptoms. If the case had no symptoms, a contact person is defined as someone who had contact with the case within a timeframe ranging from 72 h before the sample which led to confirmation was taken, to 14 days after the sample was taken. Additionally, contacts were classified according to the risk of exposure. High risk contacts (red-ring) were those who had come into unprotected face-to-face contact (within 2 m) or having been in a closed environment (e.g., household members) with a COVID-19 case for >15 min. Unprotected direct contact with infectious secretions of a COVID-19 case was also considered high risk (red-ring: these are immediate family, friends, relatives or co-workers that were more likely to have received exposure to transmission). Low risk contacts were those who had come into contact while masked, within more than 2 m or for less than 15 min. 

Oropharyngeal swabs were taken from all identified contacts and laboratory confirmation conducted using RT-PCR testing for SARS-CoV-2. Those testing negative were put under quarantine either at home or at a designated facility for 14 days from last exposure to the last confirmed case, in their cluster, while confirmed cases were isolated in individual rooms at the treatment center. Further clinical investigation and case management were conducted following the illness presentation. Data collection was led by 11 trainees of the Rwanda Field Epidemiology Training Program. They also summarized the data using a standardized line list. Collation was done by the data management desk at COVID-19 National Command post. All investigations and procedures were conducted as part of the national response under the stewardship of the National COVID-19 Taskforce. Secondary attack rates were calculated as number of cases divided by number of contacts.

## 3. Results

**Figure 1:**
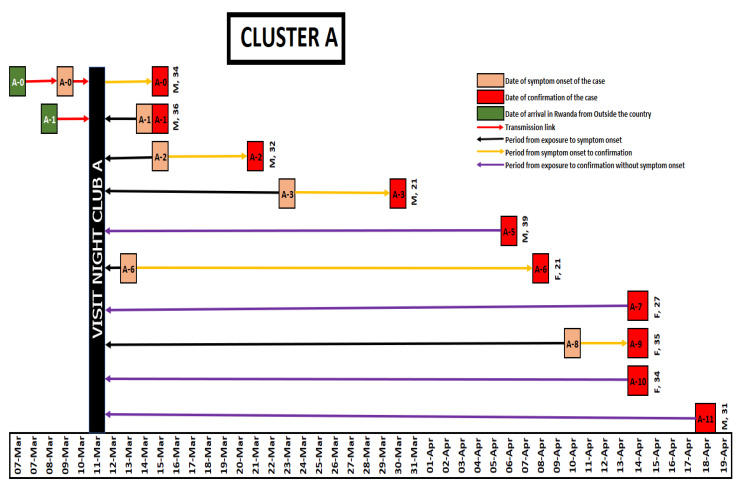
Illustration for Cluster A.

**Figure 2:**
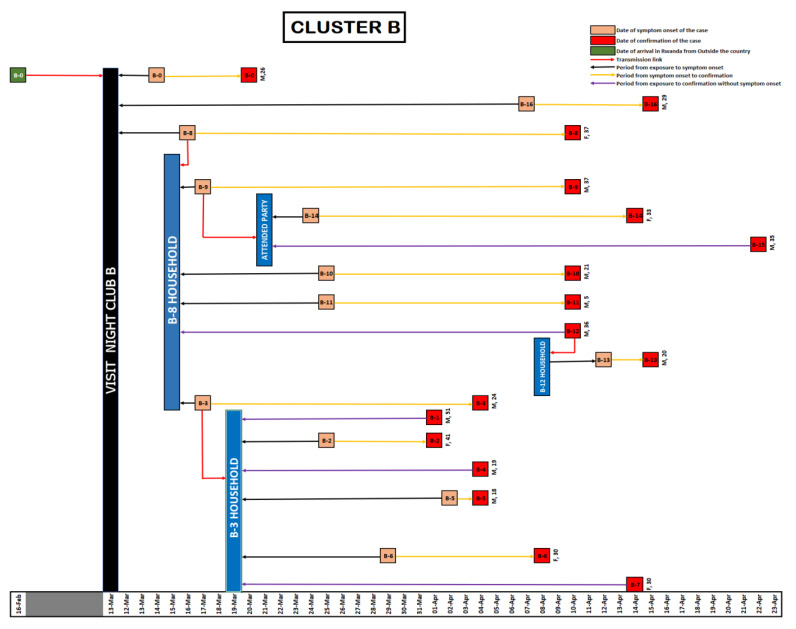
Illustration for Cluster B.

**Figure 3:**
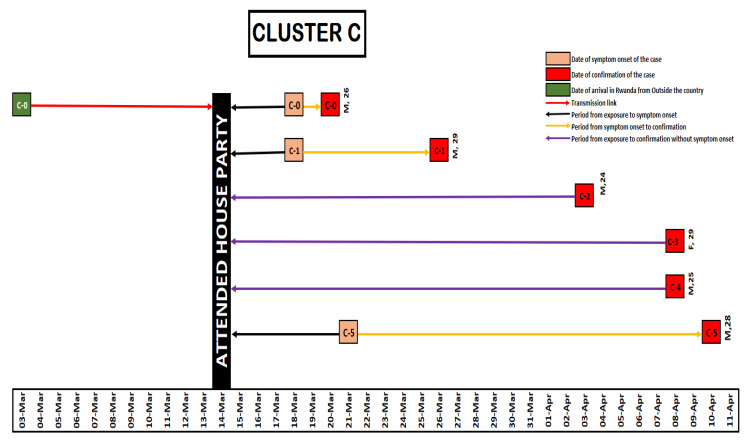
Illustration for Cluster C.

**Figure 4:**
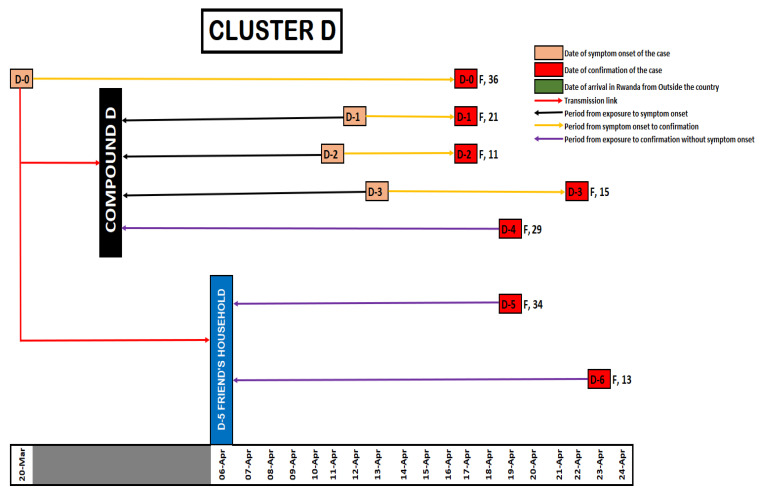
Illustration for Cluster D.

As of 22 April 2020, a total of 154 COVID-19 cases had been reported in Rwanda. Of these, 88 cases (57%) were imported through Kigali International Airport and 66 cases (43%) were locally transmitted. These cluster investigations revealed that five of the imported cases were linked to 40 locally transmitted cases in four clusters. The locally transmitted cases in these four clusters accounted for 61% of the local transmission by then. Cluster A (Night Club A) was linked to ten cases (Figure 1), Cluster B was linked to 17 cases (Figure 2), Cluster C was linked to 6 cases (Figure 3) and Cluster D was linked to seven cases (Figure 4). The detail of each cluster is provided below. 

### 3.1. Cluster A

Cluster A was connected to a popular nightclub in Kigali (labelled as Club A) and operated seven days per week before 14 March 2020. The first case (called A0) associated with this cluster was a Rwandan man (age 34 years) who had arrived in Rwanda from South Sudan on Friday 6 March 2020 and visited Club A from 11 through 13 March. On 9 March he had developed headache, fever, nasal congestion and cough. He initially self-medicated with over the counter antipyretics and was confirmed by the laboratory testing of RT-PCT as SARS-CoV-2 positive, when he presented to a health facility, on 14 March 2020.

The second case in this cluster (called case A1), was a Rwandan man (age 36 years) who arrived in Rwanda on 8 March 2020. He had travelled from Fiji, by changing two airplanes in each of the USA (John F. Kennedy International Airport, New York, NY, USA) and Qatar (Hamad International Airport, Doha, Qatar). Also, he visited Club A on 13 March together with Case A0. He developed generalized body malaise and a cough on 14 March and sought care in a health facility on 15 March after learning that case number A0 had tested positive for SARS-CoV-2. These two cases are considered to be the probable sources of infection for the Cluster A associated infections. The third case in this cluster (called A2) was a staff member in Club A who was considered as a high-risk contact. He was a Rwandan man (age 32 years) who developed flu-like symptoms including fever and cough on 15 March. He initially sought treatment in a private dispensary on 17 March 2020 before visiting a higher-level health facility on 19 March 2020 where testing confirmed COVID-19 on 21 March 2020. A total of 25 additional staff of Club A were identified to be high risk contacts of the three previous cases. All of these 25 staff were tested and 4/25 (16%) tested positive (three men and one woman). After identifying five confirmed cases (the third case in this cluster and four additional cases) among staff of Club A, further investigations were conducted to identify and test patrons who had visited the club during 1–13 March when the club was closed. Using a combination of approaches including interviews and club membership records, we found a total of 146 clients who had visited the club during the period. Among these, 93 clients were women and 53 clients were men. Three women clients of Club A were confirmed to have COVID-19. Two of them did not have symptoms at the time of the investigation. This cluster identified 264 contacts, including clients and staff of Club A and the family members of cases. Figure 1 shows the schematic illustration of the transmission links in this cluster.

### 3.2. Cluster B

On 1 April 2020, a man, aged 51 years (called case B1) and a 41 year old woman (called case B2) from one household tested positive for COVID-19. Through interviews, we initiated a list of traced contacts. Among these contacts, one family member of the confirmed cases had a travel history visiting a family in Gicumbi District (located 63 km north of the capital city, Kigali, Rwanda) on 17 March 2020 and after the visit he reported influenza-like symptoms. He self-medicated but as control measures for COVID-19 were already established, he contacted Rwanda Biomedical Center team through a toll-free number—114—and informed them that he had symptoms of COVID-19. A nasopharyngeal swab sample was collected on 2 April 2020 and he was confirmed PCR positive on 4 April 2020 (Case B3). Then his network for the period of 5 through 9 April 2020 was contacted and nasopharyngeal swab samples were collected. His family members were tested (n = 9) and four of them tested positive (labeled as Case B4, B5, B6 and B7). Furthermore, the family of five members he visited in Gicumbi District were tested and all were confirmed as PCR positive: Wife (Case B8), Husband (Case B9), Housemaid (Case B10), Child (Case B11) and their driver (Case B12) tested positive on 10 April 2020.

For the cases identified in Gicumbi District, contact tracing was conducted and identified contacts tested. On 15 April 2020, a 20 year-old contact who was sharing the same house with Case B12 was confirmed COVID-19 positive (Case B13).

The investigation of Case B9 showed that he attended a private party, at a household, on 21 March 2020 in Kigali. Among the people who attended this party, a 33 year-old woman (Case B14) and a 35 year-old man (Case B15) were confirmed as COVID-19 Positive on 14 and 22 April 2020, respectively.

As the investigation of this cluster evolved it was found that on 13 March 2020, the case B8 visited a night club, Club B, and met a man, 30 years of age, who had a travel history, returned to Rwanda on 16 February 2020 and he was confirmed positive (Case B0) on 20 March 2020. We considered this case as the probable source of infection transmission in Cluster B. In addition, on 15 April 2020 a 29 year-old Rwandan man was confirmed positive for COVID-19 (called case B16) and the investigation revealed that he previously visited the night club, Club B, and was in contact with case B8 on 13 March 2020.

All the cases cited above have a local SARS-CoV-2 infection, where the incubation period among the cluster members was 3 to 15 days (see Figure 2).

### 3.3. Cluster C

On 19 March 2020, a man, 26 years of age (Case C0) presented at the King Faisal Hospital, Kigali, with cough, fever, chest pain and body weakness. The laboratory testing confirmed SARS-CoV-2 infection on 20 March 2020. He arrived in Rwanda on 3 March 2020, travelling from Belgium. All his established local contacts since his arrival were identified and tested. On 26 March 2020, a 29 year-old man from the Netherlands elicited among contacts of case C0 was confirmed SARS-CoV-2 positive (called case C1). Case C1 arrived in Rwanda on 2 March 2020 from the Netherlands. The investigations revealed that C0 and C1 had both attended a house party on 14 March 2020. A total of 90 people who had attended the house party were identified, contacted and tested (n = 90). From those contacts, four additional cases were identified, including cases C2, C3, C4 and C5. All their contacts were quarantined, and no further cases were reported in this cluster (see Figure 3).

### 3.4. Cluster D

On 15 April 2020, a Rwandan woman, 36 years of age (labelled as D0) and living in Compound D, called the national emergency toll free number, 114. She reported a history of fever, shortness of breath and headache. A team from the national response team went to assess and decided to get her hospitalized for care and investigations. Laboratory testing for SARS-CoV-2 was positive on 17 April 2020. She has one daughter, 11 years of age (D2) and one woman, 21 years of age, working as the housekeeper (D1), who were also confirmed positive the same day. The test results for another daughter, 15 years of age (D3) was initially indeterminate but turned positive on 22 April 2020.

Two additional cases were confirmed from this cluster on 19 April including a woman, 29 years of age (labelled as D4). She lived in the same compound as case D0, and a 34 year-old woman (D5) who visited a household where case D0 had spent a whole day. On 23 April 2020, a 15 years of age daughter (D6) of case D5’s boyfriend living in the aforementioned household was also confirmed to be SARS-CoV-2 infected, through contact tracing diagnosis. However, the boyfriend’s tests, on 18 April 2020 and 24 April 2020 were negative (see Figure 4).

### 3.5. Epidemiological and Clinical Characteristics

During the period of 14 March 2020–22 April 2020 we have identified a total of 40 cases that were reported from a total of four clusters. From contact tracing, a total of 1035 contacts were identified and tested. Thirty-six locally transmitted cases (secondary attack rate = 3.5%) were identified from these four clusters. Attack rates were highest among the contacts identified as highest risk (10.2%) compared to low risk contacts (1.5%) (Table 1).

Fifteen of the 40 cases (37.5%) in the four clusters were asymptomatic. Among the symptomatic cases, mean duration from earliest date of potential exposure to onset of symptoms was 9 days (min-max 2; 29 days). Thirteen of 25 symptomatic cases (52%) had a cough. Fever and sore throat were reported by 8/24 (32%) and 4/24 symptomatic cases (16%) respectively; However, only 1/24 symptomatic cases (4%) presented with shortness of breath. No differences in clinical presentation (symptomatic vs asymptomatic) were observed across demographic characteristics.

## 4. Discussion

These in-depth epidemiological investigations were conducted to identify and characterize potential clusters of COVID-19 early in the outbreak in Rwanda. Through application of traditional epidemiological methods—including active case finding through contact tracing and testing—four clusters of accounting for the first 40/66 (61%) locally transmitted cases occurring in Rwanda within the first 40 days were identified. The investigations helped to understand transmission chains and established that the early cases were linked to imported cases. The involvement and rapid deployment of trainees and graduates from the Rwanda Field Epidemiology Training Program highlighted the importance of such programs in enhancing epidemiological workforce for outbreak response. The observed secondary attack rate in this study findings was 3.5%. This was lower compared to the secondary attack identified in other countries. A study conducted in Uganda showed the secondary rate of 13% while for two other studies conducted in China, one indicated a secondary attack rate of 49.56%, and another one with 31% [6,7]. In the four clusters, the secondary transmission was higher among those who were defined as “high-risk” contacts (labelled as a “red-ring”). These included those who lived in the same household (Clusters B and D) or who had spent a substantial amount of time at close quarters within an enclosed setting (Cluster A, B and C). The study suggests that the intensive case investigations, contact tracing and testing followed by isolation of positive cases and quarantining of contacts, may have been important factors in the early containment of COVID-19 in Rwanda. On 21 March 2020, the social-mitigation measures—including the closure of educational institutions, entertainment locations, social gatherings, government and private institutions for one week after the first case—may have prevented the rapid amplification of transmission that may have occurred. Only 66/154 locally transmitted cases (43%) had been reported in Rwanda nearly six weeks after the first case-report. The early containment measures may have helped to reduce early sustained transmission and allowed the country to put in place containment measures. Another observation in this study relates to the role of transmission from asymptomatic cases or those with mild symptoms. Among the 40 cases, 14 did not have any symptoms (35%) while the remaining 25/40 (62.5%) had minor symptoms (13 with cough, 8 with fever, 4 with sore throat, 1 with shortness of breath) by the time of the laboratory confirmation. This is slightly similar to a study conducted among early cases in Uganda where 37% did not develop symptoms [8]. However, some studies in different countries indicated that early cases had a low proportion of asymptomatic cases [9,10]. This implies that without social-distancing measures, these are people who would have been going about their lives as usual, interacting with other people and potentially initiating new chains of transmission and exacerbating the community spread of COVID-19. The surveillance measures that had been put in place, especially at health facilities, and the social mobilization that encouraged those with even minor symptoms to contact health authorities, potentially contributed to early detection of the cases followed by identification of the associated contacts.

This study is considered important in terms of reporting the SARS-CoV-2 outbreak in a low-income country in Sub-Saharan Africa. Yet, we should also mention few limitations. The testing of contacts across the country was done using Reverse Transcription Polymerase Chain Reaction (RT-PCR) assays at National Reference Laboratory. According to WHO, RT-PCR is more sensitive, however it is therefore possible that some of the negative contacts could have been early cases who were no longer shedding the virus [11]. Future investigations may need to augment testing using serological assays to identify those who may have recovered but had IgMs or IgGs against SARS-CoV-2 [12]. Additionally, viral culture was not done and the actual viral linkages could therefore not be ascertained to learn more about viral structure, replication, genetics and its effect on the host. Despite the aforementioned limitations, this study is important to the extent that documents the role of detailed epidemiological investigations, contact tracing and testing in steering containment measures early in an outbreak.

## 5. Conclusions

Gatherings in closed spaces were the main early drivers of transmission. Systematic case-investigations contact tracing and testing likely contributed to the early successes in containment of SARS-CoV-2 in Rwanda.

## Figures and Tables

**Table 1:** Attack rates of contacts of COVID-19 cases in four clusters by selected characteristics, Rwanda, 2020.

Characteristic	Number of Contacts Traced	Infected	Secondary Attack Rate
Clusters	N = 1035	N = 36	
Cluster A	264 (25.5%)	9 (25%)	3.4%
Cluster B	526 (50.8%)	16 (44.4%)	3.0%
Cluster C	90 (8.7%)	5 (13.8%)	5.5%
Cluster D	155 (15.0%)	6 (16.6%)	3.9%
Sex	N = 1035	N = 36	
Woman	376 (36.3%)	16 (44.4%)	4.3%
Man	659 (63.7%)	20 (55.6%)	3.0%
Age (years)	N = 1035	N = 36	
0–9	36 (3.5%)	1 (2.7%)	2.7%
10–19	42 (4.1%)	4 (11.1%)	9.5%
20–29	379 (36.6%)	16 (44.4%)	4.2%
30–39	538 (52.0%)	13 (36.1%)	2.4%
40–49	6 (0.6%)	1 (2.7%)	16.7%
50–59	34 (3.3%)	1 (2.7%)	2.9%
Type of contact	N = 1035	N = 36	
High risk	236 (22.8%)	24 (66.7%)	10.2%
Low risk	799 (77.2%)	12 (33.3%)	1.5%

## Data Availability

Data supporting reported results can be found through Health Management Information System (HMIS) for COVID-19: https://hmis.moh.gov.rw/covid19, accessed on 10 February 2021.
